# STRAW: Species TRee Analysis Web server

**DOI:** 10.1093/nar/gkt377

**Published:** 2013-05-09

**Authors:** Timothy I. Shaw, Zheng Ruan, Travis C. Glenn, Liang Liu

**Affiliations:** ^1^Institute of Bioinformatics, University of Georgia, Athens, GA 30602, USA, ^2^Department of Environmental Health Science, University of Georgia, Athens, GA 30602, USA and ^3^Department of Statistics, University of Georgia, Athens, GA 30602, USA

## Abstract

The coalescent methods for species tree reconstruction are increasingly popular because they can accommodate coalescence and multilocus data sets. Herein, we present STRAW, a web server that offers workflows for reconstruction of phylogenies of species using three species tree methods—MP-EST, STAR and NJst. The input data are a collection of rooted gene trees (for STAR and MP-EST methods) or unrooted gene trees (for NJst). The output includes the estimated species tree, modified Robinson-Foulds distances between gene trees and the estimated species tree and visualization of trees to compare gene trees with the estimated species tree. The web sever is available at http://bioinformatics.publichealth.uga.edu/SpeciesTreeAnalysis/.

## INTRODUCTION

Understanding phylogenetic relationships among taxa and genes is critical to the correct interpretation of many issues in biology, ranging from systematics to infectious diseases. As phylogenomic data become increasingly available, it has been hoped that the tree of life would be resolved using genome-scale data (1). One of the challenges facing phylogenomic analysis is the observation of a tremendous amount of variation in gene trees estimated from multilocus sequence data (2). This observation stimulated research on the estimation of species-level phylogenies (i.e. species trees) by taking into account variation at the level of individual genes (3–6).

The past few years have witnessed a fast expansion of species tree reconstruction methods. Phylogenetic programs MP-EST (7), STAR (8), NJst (9) developed under the coalescent model (3) have been widely used for estimating species-level phylogenies (10). A major strength of these three methods is that they are computationally tractable, even for data sets that are large (10), and thus are amendable to making an open resource for the research community with only modest hardware requirements. Many additional phylogenetic programs have been developed for species tree reconstruction, such as *BEAST (11), BEST (4) and STEM (5), but these methods are computationally intensive and thus are not amenable to an open resource built on modest hardware.

MP-EST, STAR and NJst use gene trees estimated from DNA sequence data to infer species trees. Uncertainty of the estimated gene trees is incorporated in estimation of species trees using bootstrap techniques. In the MP-EST method, species trees are estimated from a collection of rooted gene trees by maximizing a pseudo-likelihood function of triplets in the species tree. The STAR method uses average ranks of gene coalescence times to build species trees from a set of rooted gene trees. The STAR method is implemented by building a Neighbor Joining (NJ) tree (12) from a distance matrix in which the entries are twice the average ranks across gene trees. In contrast to MP-EST and STAR, the NJst method is able to use unrooted gene trees to infer the phylogenies of species. All three methods can quickly estimate species trees even for large-scale phylogenomic data and they are statistically consistent under the coalescent model (13). The three methods are fairly robust to a limited amount of horizontal transfer as well as deviations from a molecular clock because some small values of coalescence times due to horizontal transfer or rate variation in particular genes do not have major effects on the average ranks and the frequencies of gene tree triplets when the number of genes is moderate or large (10). A comparison of the three methods is given in Table 1.

**Table 1.** Comparison of three coalescent-based species tree reconstruction methods available to users of STRAW

	MP-EST	STAR	NJst
Input	Rooted binary gene trees	Rooted binary gene trees	Unrooted binary gene trees
Can estimate topology?	Yes	Yes	Yes
Can estimate branch lengths?	Yes	No	No
Branch units	Coalescence units	NA	NA
Runtime (50 taxa 100 genes)	1656 s	9 s	46 s
Programming language	**C**	R	R
Reference number	8	9	10

## WEB SERVER

The Species TRee Analysis Web server (STRAW) provides a user-friendly web interface specifically for MP-EST, STAR and NJst analyses. STRAW consists of a series of species tree algorithms and input data processing and analysis visualization tools including (i) rooting gene trees with outgroup species, (ii) building STAR, MP-EST, NJst trees, (iii) comparing gene trees with the estimated species tree and (iv) bootstrap analyses.

The MP-EST algorithm is written in the C programming language and is available as a standalone binary at http://code.google.com/p/mp-est/, whereas STAR and NJst are implemented in an R package (Phybase) available at http://code.google.com/p/phybase/. The STRAW web server is implemented through a combination of php, perl and java programs. The front end of the server is implemented through standard HTML markup language using javascript and the jQuery library. The server runs as a dedicated Linux machine with eight 2.8 GHz Intel i7 processor cores and 8 GB of RAM.

### Server input and workflow

For the MP-EST and STAR methods, the input gene trees must be bifurcating rooted trees in Newick format, for example, the ML trees generated from PHYML (14), RAXML (15) or PHYLIP (16), and rooted with the outgroup species. The input gene trees for NJst are either rooted trees or unrooted trees. The MP-EST and STAR methods can handle missing taxa in gene trees. Thus, it is fine if some genes for some of the species are missing in the input data. The user must provide a species–allele table to indicate the relationship between alleles and species (i.e. which alleles belong to which species). For example, the following gene trees have taxa A1–6.

(((((A1:0.1,A2:0.7):0.1,A3:0.5):0.1,A4:0.2):0.9,A6:0.4):0.1,A5:0.8);
(((((A1:0.2,A2:0.2):0.1,A4:0.3):0.1,A5:0.7):0.2,A3:0.1):0.1,A6:0.7);
(((((A2:0.4,A1:0.1):0.1,A6:0.7):0.1,A3:0.8):0.1,A5:0.1):0.1,A4:0.6);

Suppose A1 and A2 were sampled from Human, A3 and A4 were sampled from Ape, A5 was sampled from Gorilla and A6 was sampled from Chimpanzee. Then the species–allele table should be (row order is arbitrary)

**Table:** 

Human	2	A1 A2
Ape	2	A3 A4
Gorilla	1	A5
Chimpanzee	1	A6

**Figure 1.**
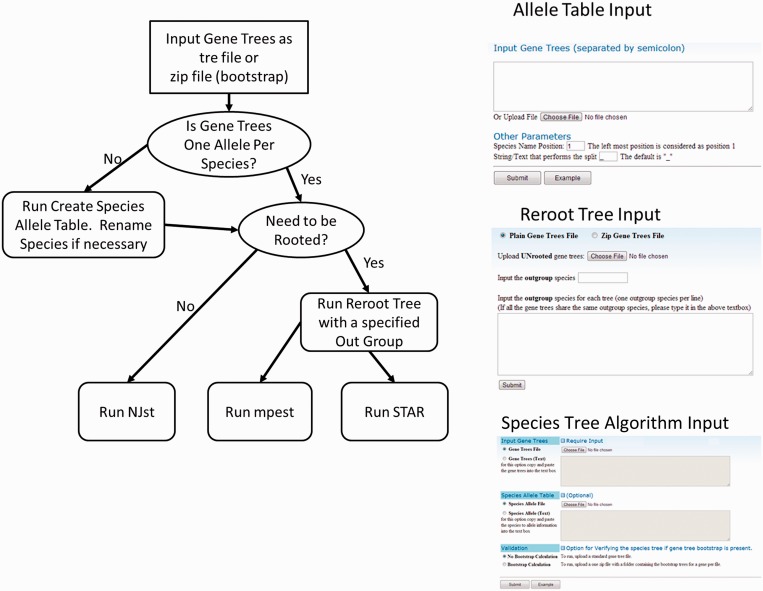
Workflow for Species Tree Construction. To run the species tree algorithm, Newick gene trees and species allele information needs to be provided. We provide the user the capability to create a species to allele table. For MP-EST and STAR, gene trees need to be rerooted to particular outgroup before running.

Each line specifies ‘the species name’, ‘number of alleles’ and ‘the names of the alleles’. To assist users with construction of species allele tables, the program SpeciesAlleleTableCreator can generate an example input file, which assumes a one to one correspondence between species to allele information. The SpeciesAlleleTableCreator program is designed for the user to edit the allele information before passing it to the species tree algorithms (Figure 1). Under the circumstance that no Species Allele Table is provided to the species tree algorithms, the program will assume the name for each allele as individual species (one to one correspondence between species and alleles). For MP-EST and STAR methods, a rooted tree is required as input. Thus, we provide functionality for rooting the tree via the program RerootTreeInput (Figure 1). The user will need to indicate the outgroup for rooting the tree. Bootstraps of gene trees can be uploaded to the server through a zip folder. Each file in the zip folder contains bootstrapped gene trees for a single gene. We implement a multilocus bootstrap method based on Seo *et al.* (17).

### Server output

**Figure 2.**
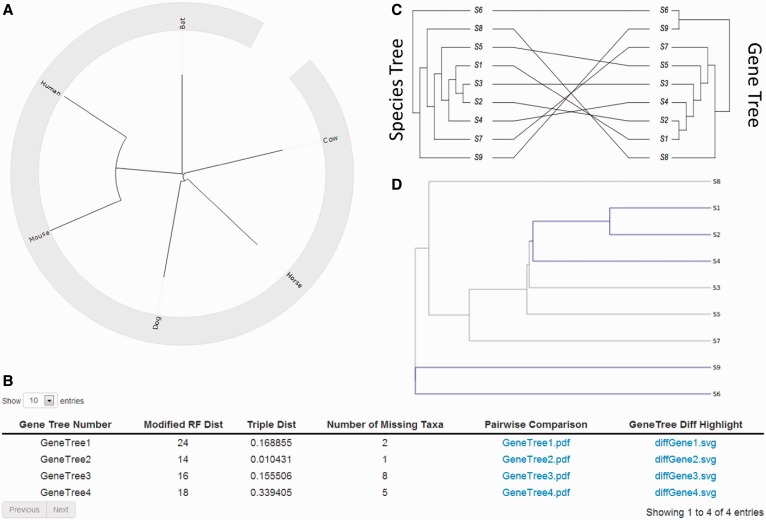
Species tree and gene tree for the Jamaican Fruit Bat compared with human, mouse, cow, horse and dog. (**A**) A NJst tree from 2378 gene loci placing bats sister to Perissodactyla, Cetartiodactyla and Carnivora. (**B**) A table is presented listing the RF distance, triple distance and number of missing taxa. (**C**) We also place gene tree and species tree side by side with matching node tip mapped to each other. (**D**) For each gene tree, mismatching branches (compared with species tree) are colored blue, and similar branches are colored gray.

The output of the STAR, MP-EST and NJst analyses includes the estimated species trees in Newick format, which are also presented to the user via a web page containing a circular phylogenetic tree generated by jsPhyloSVG (18). The SVG phylogenetic tree is downloadable by the user for publication purposes. Figure 2A is an example showing the NJst-generated species tree from data of Shaw *et al.* (19), including 2378 gene loci for bat, cow, dog, horse, human and mouse. As part of the output, we generate a report to compare each gene tree against the estimated species tree (Figure 2B). Within the report, we computed the Robinson and Foulds (RF) topological distance (20) between gene trees and the estimated species tree. The RF topological distance measures the tree similarity; the lower the number the greater the similarity between the gene tree and the estimated species tree. We modified the RF distance to allow missing taxa by first finding the common taxa that appear on both trees, then both trees are pruned to have only the common taxa and finally the RF distance is calculated for the two pruned trees with the same set of taxa. We also include gene tree species tree comparison plot. The gene tree species tree comparison plot uses function cophyloplot from an R package APE (21) and plot two trees face to face with links between the tips (Figure 2C). For MP-EST we calculate triple distance between gene trees and the estimated species tree. The server provides an additional functionality of comparing the gene tree and species tree using ‘compareInter2tips’ Bio.Python (22). Gene trees with conflicting branches (with species tree) are colored blue, and branches that are the same are colored gray (Figure 2D).

## CONCLUSION

STRAW is a useful web application for estimating species trees. The server provides a user-friendly web interface for three coalescent programs (MP-EST, STAR, NJst), along with phylogenetic tools for visualizing trees, calculating tree distances and rooting gene trees. Our web server tools are most useful in species with disagreeing gene trees and it is able to make significant contribution in resolving the systematic problem of heterogeneity in gene trees in terms of topology or branch length. Through the different web server results, we can help develop hypotheses for distinguishing deep coalescence and branch length heterogeneity for both gene trees and species trees alike. The server does not require registration and provides open access to the research community.

## FUNDING

The web server is graciously maintained on hardware of the University of Georgia’s College of Public Health. Funding for open access charge: Start-up funds from University of Georgia for early career promotion (to L.L.); National Science Foundation (DEB-1242241 and DEB-1136626 to T.C.G.; DMS-1222745 to L.L.).

*Conflict of interest statement.* None declared.
